# Position Statement from the German Biobank Alliance on the Cooperation Between Academic Biobanks and Industry Partners

**DOI:** 10.1089/bio.2019.0042

**Published:** 2019-08-12

**Authors:** Ronny Baber, Michael Hummel, Roland Jahns, Magdaléna von Jagwitz-Biegnitz, Romy Kirsten, Corinna Klingler, Sara Y. Nussbeck, Cornelia Specht

**Affiliations:** ^1^ Institute of Laboratory Medicine, Clinical Chemistry and Molecular Diagnostics, University of Leipzig Medical Center, Leipzig, Germany.; ^2^ Leipzig Medical Biobank, University Leipzig, Leipzig, Germany.; ^3^ Institute of Pathology, Charité—Universitätsmedizin Berlin; and Central Biobank Charité (ZeBanC), Berlin, Germany.; ^4^ German Biobank Node, Charité- Universitätsmedizin Berlin, Berlin, Germany.; ^5^ Interdisciplinary Bank of Biomaterials and Data Würzburg (ibdw), University and University Hospital Würzburg, Würzburg, Germany.; ^6^ Biobank of the National Center for Tumor Diseases (NCT) Heidelberg, Heidelberg, Germany.; ^7^ QUEST—Center for Transforming Biomedical Research, Charité - University Medicine Berlin, Berlin Institute of Health (BIH), Berlin, Germany.; ^8^ University Medical Center Göttingen, UMG Biobank, Göttingen, Germany.

**Keywords:** cooperation, industry, academic biobanks, German Biobank Alliance, position article

## Abstract

Under the umbrella of the German Biobank Node (GBN), 11 biobanks and two IT development centers are funded by the Federal Ministry of Education and Research (BMBF) to work together in the German Biobank Alliance (GBA). Their common aim is to make existing biomaterials hosted by different biobanks nationally and internationally available for biomedical research. This position article reflects and summarizes contributions and comments made during a GBA workshop, on the cooperation between academic biobanks and pharmaceutical and diagnostics companies that took place in Leipzig on the 21st of June 2018. It documents key points agreed on by all participating biobanks during the workshop thereby addressing several of the challenges identified. Although there are various possibilities for cooperation between academic biobanks and industry, this position article focuses exclusively on projects where academic biobanks give access to their biosamples and related data to industry partners. In doing so it considers the general conditions/framework and procedures in the German biobanking environment and raises ethical, legal, and procedural issues to be addressed when initiating such collaborations. It intends to furnish a basis for further activities to foster cooperation with industry and to push an overarching national coordination process. The final aim is to develop GBN-recommendations. Of course, many hospitals already have clear regulations on collaboration(s) with industry partners. These naturally take precedence for the GBA biobanks. However, where interest exists, GBN/GBA recommendations could help to induce changes to existing local policies nonetheless.

## Introduction

### Definition and motivation for cooperation with industry: benefits and challenges

Cooperation with industry comprises both types of projects in which (1) academic biobanks provide the pharmaceutical or diagnostics industry^†^ with existing biosamples and related data under appropriate conditions, and projects in which (2) biobanks merely take care of the sample logistics (including storage)—for example, in the frame of clinical trials. Although the first of these options is often viewed critically (depending on the prevailing conditions/framework), the second one merely using the biobank infrastructure is not.^‡^ This position article, therefore, focuses exclusively on cooperation where academic biobanks make existing biosamples and related data available to industry partners.

To develop recommendations for cooperation with industry in the latter sense, the potential benefits but also the risks of such cooperation must be analyzed. In principle, cooperation with industry harbors considerable potential, as both the development of therapeutics and the validation of diagnostics strongly depend on human biosamples and data. In the case of cooperation with industry, it is generally desirable that a majority of results and data generated by the industry partner(s) are returned to the academic donor institutions to enrich the academic database for future research projects. Thus, cooperation with industry would provide benefits to medical research and, in the long term, ultimately also to the donors/patients.

Academic biobanks may also benefit significantly from cooperation with industry, as

(1) joint publications may arise from common projects;
(2) successful joint (industry) projects enhance the reputation and visibility of academic biobanks;
(3) cooperation with industry represents an additional external source of funding for biobanks and may thus contribute to biobanks' long-term sustainability; and
(4) collaboration and exchange with industry may boost mutual knowledge and experience.

At the same time, however, cooperation with industry also entails certain risks and challenges that require different solutions and a defined framework.

## General Arrangements for Cooperation with Industry

German Biobank Alliance (GBA) biobanks agreed unanimously that an unrestricted transfer of biosamples and related data to industry is unacceptable. Any release and transfer of biosamples and related data to third parties always require a written proposal with a project description outlining the planned use of the requested biosamples and/or data. This description also forms the basis for the approval by an independent competent (i.e., in Germany, appointed according to state legislation) medical ethics committee. Moreover, almost all academic biobanks have a “Use and Access Committee” (UAC) that finally decides on the release and transfer of biosamples and/or data.

The following scenarios for cooperation with industry are conceivable under the aforementioned procedural and ethical framework:

Scenario 1: The industry partner may get access to biosamples and related data within the scope of research cooperation, ideally documented by a subsequent authorship in scientific publication. In addition, the respective (advising) ethics committee must approve the collaborative project and the biobank UAC must give its permission.
Scenario 2: Under certain conditions (e.g., no specific research interest of the biobank or its hosting institution in the given topic), the industry partner may also get access to biosamples and related data as a service without explicit research cooperation. However, this will equally require a proposal/project description to be submitted both to the advising ethics committee and the UAC of the respective biobank.

Industry ultimately aims at using biosamples and related data from academic biobanks to develop products that are suitable for therapeutic and/or diagnostic applications. Commercial companies generally rely on their products often returning a handsome profit that can be reinvested in new developments. The direct sharing of such profits with the biobank–donors/patients is neither feasible nor does make sense. However, academic biobanks can ask for a compensation for their own expenses.^§^ The “broad consent” template developed by the permanent Working Party of the German Medical Ethics Committees (AK-EK) states: “You will not receive any remuneration for donating your biomaterials and/or data for medical research purposes. […] The biobank may charge a reasonable compensation for providing biomaterials and/or data to users.” However, there is no clear definition of what “reasonable” compensation means.

The concept of “reasonable compensation” for expenses remained ambiguous during the workshop. However, the GBA biobanks agreed that this compensation should in principle be confined by the real full costs, meaning that such financial compensation should not exceed the full costs. Coverage of the full costs incurred by the biobank was considered a suitable guideline for the concept of “reasonable” compensation and is an argument against a possible reproach of unfair competition. However, a pure sale of voluntarily donated biomaterials and/or related data with clear profit intentions was deemed ethically unallowable. Services provided for external partners and revenues from research cooperation with external partners (not affiliated to a university/university hospital) are also subject to value-added tax and institutional overhead.

## Impact of Cooperation with Industry on the Willingness to Donate

For academic biobanks, the fundamental question also arises of the impact that cooperation with industry partners might have on the willingness of patients and study participants to donate. Will patients potentially withdraw their consent when they learn who received their biosamples and/or data, despite having consented to the possibility that private enterprises might use their samples/data? The question also arises of how cooperation with industry could affect patient donation behavior in terms of the long-term strategic orientation: does cooperation with industry have a positive or negative impact on patients' trust in academic biobanks? Might it decrease the willingness of certain patients/study participants to donate?

Whether and how these risks have an effect largely depends on potential donors' behavior and attitude. To gain a realistic assessment of the donors view here it is essential to initiate a dialogue with potential donors. This dialogue is important to learn about donors' needs and expectations—as a basis for their adequate consideration. It may also serve to explain the urgent need for a tight cooperation between academic and industry partners and, thus, overcome prejudices against industry.

## Data Sharing Between Industry and Academic Biobanks

In the case of cooperation with industry, it is generally desirable that a majority of results and data obtained from the analysis of furnished biosamples are returned to the academic donor institutions to enrich the academic database for future research projects. One GBA biobank has already agreed with its industry partner(s) that the results and data obtained by using its biosamples shall be systematically returned to be available for future research projects. However, there was some criticism that this principle may deter industry partners, because the results of industrial research projects cannot always be made public at an early R&D stage (probably constituting a competitive disadvantage for industry partners with regard to intellectual property and/or patent rights). Another GBA biobank has signed a confidentiality agreement for such cases and grants the data-generating companies a veto right before data are passed on to other projects. This approach could also serve as a generic model for cooperation with industry.

A further challenge is how to deal with the competitive situation between academic biobanks, which only request compensation for expenses, and biobanks, which in addition also require the industry partner to return their research results. Data sharing would entail that research data generated by the industry partner are returned to the respective biobank, which might represent a potential obstacle for cooperation with academic biobanks.

## Summary and Outlook

This position article highlights key issues and possible solutions to the challenges of cooperation between academic biobanks and industry. It intends to provide a basis for further bilateral activities toward cooperation with industry and to induce an overarching national coordination process.

- Cooperation with industry to support their developments with biosamples and related data is important and deserves support.
- This cooperation should be preferably performed as research collaboration for mutual benefit.
- Cooperation without explicit research collaboration is feasible, however, needs to follow the same rules (project description, ethics approval, and UAC permission).
- Compensation of expenses is based on full cost calculation (not including taxes and institutional overhead).
- Sharing of data derived from the biosamples provided should be returned to the academic donor institutions under defined conditions to accelerate biomedical research.

Whether and how cooperation between academic biobanks and industry affects the willingness of patients/study participants to donate their biosamples and related data largely depends on attitude and background knowledge of donors. The German Biobank Node (GBN) currently initiates a dialogue with (potential) donors to get more insights in this regard and to raise understanding for the importance to support industry with biosamples and data.

## Footnotes

^†^ Unless explicitly stated otherwise, in the present article the term “industry” comprises both the pharmaceutical and the diagnostics industries.

^‡^ It is important to note that this type of cooperation could also be viewed critically when storage space at biobanks runs short. This challenge must then be addressed by a storage strategy for the respective biobank.

^§^ From an ethical perspective, the question of “reasonable” compensation also raises the question of patient reimbursement. This is not discussed here further, although, since at present patients basically donate their samples and are not involved in the commercial or patent use of their biosamples and related data.

